# TFKT V2: task-focused knowledge transfer from natural images for computed tomography perceptual image quality assessment

**DOI:** 10.1117/1.JMI.12.5.051805

**Published:** 2025-05-28

**Authors:** Kazi Ramisa Rifa, Md Atik Ahamed, Jie Zhang, Abdullah Imran

**Affiliations:** University of Kentucky, Lexington, Kentucky, United States

**Keywords:** image quality assessment, computed tomography, transfer learning, low dose, Swin Transformer

## Abstract

**Purpose:**

The accurate assessment of computed tomography (CT) image quality is crucial for ensuring diagnostic reliability while minimizing radiation dose. Radiologists’ evaluations are time-consuming and labor-intensive. Existing automated approaches often require large CT datasets with predefined image quality assessment (IQA) scores, which often do not align well with clinical evaluations. We aim to develop a reference-free, automated method for CT IQA that closely reflects radiologists’ evaluations, reducing the dependency on large annotated datasets.

**Approach:**

We propose Task-Focused Knowledge Transfer (TFKT), a deep learning–based IQA method leveraging knowledge transfer from task-similar natural image datasets. TFKT incorporates a hybrid convolutional neural network–transformer model, enabling accurate quality predictions by learning from natural image distortions with human-annotated mean opinion scores. The model is pre-trained on natural image datasets and fine-tuned on low-dose computed tomography perceptual image quality assessment data to ensure task-specific adaptability.

**Results:**

Extensive evaluations demonstrate that the proposed TFKT method effectively predicts IQA scores aligned with radiologists’ assessments on in-domain datasets and generalizes well to out-of-domain clinical pediatric CT exams. The model achieves robust performance without requiring high-dose reference images. Our model is capable of assessing the quality of ∼30 CT image slices in a second.

**Conclusions:**

The proposed TFKT approach provides a scalable, accurate, and reference-free solution for CT IQA. The model bridges the gap between traditional and deep learning–based IQA, offering clinically relevant and computationally efficient assessments applicable to real-world clinical settings.

## 1 Introduction

Computed tomography (CT) is one of the most widely used imaging modalities for disease diagnosis, treatment planning, and surgical guidance, with over 80 million CT scans performed annually in the United States. Despite its diagnostic benefits, CT accounts for a significant portion of ionizing radiation exposure, raising patient safety concerns.^1^ Minimizing radiation exposure is essential due to its associated health risks. However, CT image quality heavily depends on X-ray radiation exposure, with higher doses producing better-quality images by reducing quantum noise.^2^ Lowering the radiation dose introduces quantum noise and artifacts, which obscure important diagnostic details and compromise diagnostic accuracy.^3^^,^^4^ Therefore, reliable metrics for CT image quality assessment (IQA) are essential to balance diagnostic image quality and minimal radiation exposure.

Traditionally, image noise has been used as an IQA metric, working well for filtered back projection. However, it becomes challenging with iterative reconstruction techniques, both image noise amplitude and texture change, reducing its effectiveness. Other common IQA metrics, such as peak signal-to-noise ratio, structural similarity index measure, and mean squared error, have also been used but fall short in clinical applications. These metrics rely on pixel-level differences and often assume the availability of high-dose reference images, which are impractical in clinical practice.^5^ Furthermore, they lack sufficient correlation with radiologists’ subjective evaluations, limiting their reliability in assessing CT image quality. Task-specific IQA metrics, such as lesion detectability,^6^ anatomical detail visibility,^7^ and diagnostic accuracy,^8^^,^^9^ have also been proposed. Although these metrics are more clinically relevant, they come with advantages and disadvantages. On the positive side, task-specific metrics align better with radiologists’ expectations, increase focus on diagnostic tasks, and provide context-aware assessments. However, they are complex, difficult to generalize, and challenging to implement in routine clinical workflows due to variability in tasks and patient-specific factors.^4^

To address these limitations, artificial intelligence (AI)-based IQA metrics have emerged as promising solutions by leveraging deep learning models. AI-driven methods based on convolutional neural networks (CNNs) and transformers have shown potential in CT image quality assessment, as demonstrated by the 2023 Grand Challenge on low-dose computed tomography perceptual image quality assessment (LDCTIQA). We propose a task-focused knowledge transfer (TFKT) approach for data-efficient IQA prediction. This method transfers knowledge from natural image IQA tasks, where high-quality images serve as references. By applying controlled distortions, lower-quality versions of natural images can be generated, enabling the calculation of mean opinion scores (MOSs) based on human feedback. At the beginning of our model, we use a CNN-based architecture as it efficiently captures local spatial details and is well-suited for feature extraction. However, CNNs struggle with modeling long-range dependencies due to their focus on small local regions. To address this, we then incorporate a transformer-based architecture to better capture global context. Our proposed approach is based on a combination of innovative task-specific pre-training and architectural modifications to the transformer to achieve better performance. With the modifications, our proposed model outperforms a number of baseline and state-of-the-art methods in predicting IQA scores from CT images.

Our proposed method, TFKT V2, leverages pre-trained models on natural images followed by fine-tuning the LDCTIQA dataset. This approach aligns well with radiologists’ evaluations (Table 1) and eliminates the need for high-dose reference images. For natural images, we use the Konstanz artificially distorted image quality database (KADID-10k) dataset,^10^ which is a widely used benchmark for image quality assessment, containing images with various distortions at different levels to evaluate the perceptual quality of images with MOS as IQA. We introduce a hybrid CNN–transformer model designed to extract meaningful features from both global image structures and local textures, enhancing prediction accuracy. Through this task-similar transfer learning approach, our model effectively bridges the gap between traditional and AI-based IQA methods, making CT image quality assessment more reliable, scalable, and clinically applicable.

**Table 1:** Image scoring criteria for the 2023 low-dose CT image quality assessment (LDCTIQA) grand challenge.

Score	Quality	Diagnostic quality criteria
0	Bad	Desired features are not shown
1	Poor	Diagnostic interpretation is impossible
2	Fair	Suitable for compromised interpretation
3	Good	Good for diagnostic interpretation
4	Excellent	Anatomical features are clearly visible

Our specific contributions in the present paper are summarized as follows:

• a novel task-specific transfer learning approach with a hybrid CNN–transformer for assessing low-dose CT image quality leveraging natural images
• a modified Swin Transformer using a combination of multi-layer perception (MLP) and Kolmogorov–Arnold network (KAN) with the cross-attention mechanism
• extensive experimentation demonstrating the effectiveness of the proposed approach using different performance metrics and ablation studies
• introduce a model that minimizes inference time and enhances feasibility for real-time clinical application
• clinical validation of the proposed IQA method using clinical images of pediatric CT exams.

Our Previous Work: This paper is an extension of our paper, task-focused knowledge transfer from natural images for CT image quality assessment, to be presented at the 2025 SPIE Medical Imaging Conference.^11^ It substantially expands upon the previous work by providing a comprehensive literature review, architectural modifications, extensive experimentation demonstrating improved results, a more detailed description of methods, expanded results discussion, and additional figures and visualizations. The improvement comes from our newly modified architecture, enabling more robust and accurate CT image quality assessment.

## 2 Related Work

We carefully review the latest developments in the following topics related to our work: transfer learning, Kolmogorov–Arnold networks, and image quality assessment.

### 2.1 Transfer Learning

Transfer learning leverages knowledge gained from solving a problem in one domain (source) to improve performance on a different domain (target) problem. This strategy is particularly valuable when data for the target domain is limited or expensive to obtain.^12^ Instead of training models from scratch, transfer learning leverages large-scale pre-trained models to effectively capture generalizable patterns, which are then fine-tuned or adapted to more specific tasks. Considering the scarcity of annotated data in medical imaging, transfer learning can be a viable alternative to traditional training from scratch. It is evident that fine-tuning generally has better outcomes compared with full training across various medical imaging applications.^13^

Recent advancements have further underscored the impact of transfer learning in medical imaging. Huang et al.^14^ introduced fine-grained prompt tuning, a transfer learning framework designed to efficiently handle high-resolution medical image classification tasks. Their approach addresses significant challenges in medical imaging, such as memory limitations and the need for precise feature extraction. By keeping the weights of extensive pre-trained models fixed and adding a lightweight auxiliary network with specific fine-grained prompts, their technique greatly lowered memory consumption while preserving top-tier performance across multiple medical datasets. Zhang et al.^15^ demonstrate that transfer learning effectively leverages a pre-trained model to enhance segmentation on smaller clinical datasets, addressing the challenge of limited training data availability. Aghelan and Rouhani^16^ highlight the utility of transfer learning for improving generative adversarial network (GAN)-based super-resolution in medical imaging, showcasing its ability to compensate for limited dataset sizes and improve model convergence.

Saeed et al.^17^ proposed a transfer learning strategy using meta-reinforcement learning, demonstrating efficient adaptation of task-specific IQA models to new definitions of image quality with minimal expert-labeled data, thus enhancing model adaptability and reducing reliance on extensive labeled datasets. Saeed et al.^17^ proposed a transfer learning approach via meta-reinforcement learning, enabling IQA models to adapt efficiently using minimal expert-labeled data. On the other hand, Yang et al.^18^ introduced a transitive transfer learning framework for no-reference IQA, bridging unrelated source and target domains using an auxiliary domain and task. Transfer learning has been combined with artifact simulation to reduce manual annotation efforts yet achieve robust MRI quality assessment.^19^ Shan et al.^20^ leveraged transfer learning from a pre-trained two-dimensional (2D) model to develop a three-dimensional convolutional encoder–decoder network for low-dose CT image denoising. Automatic CT image quality control with robust diagnostic performance has been demonstrated via deep transfer learning.^21^ Matsoukas et al.^22^ explored the factors that contribute to the success of transfer learning in medical imaging, focusing on feature reuse and domain-specific adaptation. Their findings demonstrate that transfer learning is particularly effective when the source and target domains share similarities and that smaller datasets benefit significantly from pre-trained models.

This reinforces the importance of transfer learning in medical tasks where data scarcity and domain complexity demand efficient use of pre-trained features. However, most of the models leverage transfer learning between source and target domains that share similarities, the application of transfer learning for the same task across significantly different domains remains relatively underexplored.

### 2.2 Kolmogorov–Arnold Network

The use of advanced regression models in medical imaging has become increasingly critical as imaging data complexity grows. The KAN^23^ introduces a new regression approach using learnable activations on edges instead of fixed ones on nodes unlike MLPs. KANs address the weaknesses of MLPs, particularly in handling high-dimensional regression tasks. The Kolmogorov–Arnold representation theorem is incorporated in KANs to improve efficiency and generalization in models. Activation functions created via splines help capture detailed compositional structures and refine univariate functions. Thus, KANs can be highly suitable for medical imaging tasks where regression models need to identify and analyze intricate patterns in the data. One of the key strengths of KANs is their ability to tackle the curse of dimensionality while preserving accuracy and interpretability.

KANs have also demonstrated superior performance in tasks involving sparse or compositional structures. This makes them particularly valuable for regression tasks in medical imaging where high accuracy and scalability are crucial. Vaca-Rubio et al.^24^ highlighted the capability of KANs to handle time-dependent variations in imaging data, leveraging their compositional structure to forecast anomalies and trends accurately. With spline-parameterized activations and edge-based modular design, the representation capability of KANs is enhanced for imaging tasks.^25^

Liu et al.^23^ demonstrated that KANs are well-suited for scientific tasks involving modular structures and symbolic formula extraction. They effectively handle relationships in high-dimensional data while preserving interpretability and adaptability. This makes KANs applicable to domains requiring complex function decomposition. As KANs combine mathematical precision with practical efficiency, they could play a key role in enhancing regression performances in medical imaging.

### 2.3 Image Quality Assessment

A growing body of IQA research has recently emerged IQA using deep learning techniques for automated and no-reference IQA.^2^^,^^5^^,^^26^^–^^30^

Imran et al.^2^ performed a joint estimation of IQA and noise level. Although the image quality assessment task benefits from self-supervised information of the CT images, this approach utilizes an artificial noise generation procedure. Another study^31^ demonstrates CT IQA by first leveraging full-reference images for training CNN which lacks complete reference–free quality assessment. However, these methods either use proxy IQA scores—lacking direct clinical relevance or pseudo-labeling—lowering confidence for potential clinical usage. Another challenge is that most existing studies focus on CT images affected by singular types of artifacts, such as low-dose noise, view aliasing, metal artifacts, and scattering. Real low-dose CT images often exhibit a combination of these artifacts. A recently compiled LDCTIQA dataset exhibits a range of complex noise and artifacts in CT images as well as perceptual scores provided by expert radiologists.^32^

The availability of the public LDCTIQA dataset has enabled the prediction of radiologists’ IQA scores directly in a reference-free manner. Recent developments along with the top-ranked algorithms in the LDCTIQA challenge include models based on CNN, Vision Transformer (ViT)/Swin Transformer, and hybrid architectures.^5^^,^^8^^,^^27^ Song et al.^27^ proposed MD-IQA uses multi-scale distribution regression to improve robustness and reduce prediction uncertainty. However, the performance of MD-IQA is limited to unstable training and quality of labels which may lead to poor out-of-domain generalization. In addition, MD-IQA relies on a large number of unlabeled data which can be challenging to obtain in some settings. Another method D-BIQA^5^ predicts IQA scores by algorithmically generating reference images. Although the IQA performance is improved to some extent, D-BIQA still requires reference images for training the image synthesis model which poses additional challenges.

Although leveraging ImageNet^33^ weights in the IQA task can be helpful,^34^ it is not sufficient as the task is different (i.e., ImageNet’s task is object classification in natural images, whereas IQA focuses on assessing image quality based on distortions, noise, and perceptual clarity). Considering the limited number of labeled samples in the LDCTIQA dataset, transfer learning could be a viable approach to building a deep learning-based IQA prediction method. However, the substantial differences in task and domain may advise against knowledge transfer from natural to medical images.

## 3 Methods

### 3.1 Hybrid Architecture

We propose a hybrid architecture consisting of CNN and a transformer model to extract meaningful features from both global image structures and local textures and predict the IQA score effectively. Our model leverages EfficientNet as the CNN backbone and modified Swin Transformer (Swin-KAT) as the transformer backbone. Then, the CNN backbone is integrated with the transformer backbone via a connector.^11^

### 3.2 Preliminaries

#### 3.2.1 EfficientNet

EfficientNet^35^ is a convolutional neural network architecture designed to optimize both accuracy and efficiency using a compound scaling method to balance network depth, width, and resolution. EfficientNet demonstrates prominent performance across various vision tasks, including classification and transfer learning. EfficientNetV2,^36^ an advancement of the original EfficientNet, incorporates architectural improvements and optimized training procedures to achieve even better performance. It employs a combination of depthwise convolutions, fused operations, and a progressive learning strategy to reduce training time and improve efficiency. These characteristics can be quite beneficial for CT image quality assessment, where analyzing fine-grained patterns with computational efficiency is crucial.

#### 3.2.2 Swin Transformer

The Swin Transformer,^37^ introduced as a hierarchical Vision Transformer, has established itself as a versatile backbone for deep learning tasks. It addresses the limitations of traditional transformers in handling high-resolution images by introducing a shifted windowing mechanism. This mechanism achieves linear computational complexity while maintaining strong performance across tasks such as image classification, object detection, and semantic segmentation. This design enables efficient feature extraction at multiple scales, making the Swin Transformer a versatile backbone for a variety of vision tasks. Building on this foundation, Swin Transformer V2^38^ introduced enhancements that address training instability and improve adaptability to high-resolution images. Key modifications included residual post-normalization, scaled cosine attention, and a novel log-spaced continuous position bias, enabling scaling up to 3 billion parameters and processing resolutions up to 1536×1536. These improvements make Swin Transformer V2 a robust choice for tasks requiring fine-grained analysis, including medical imaging.

### 3.3 Problem Formulation

**Fig. 1:**
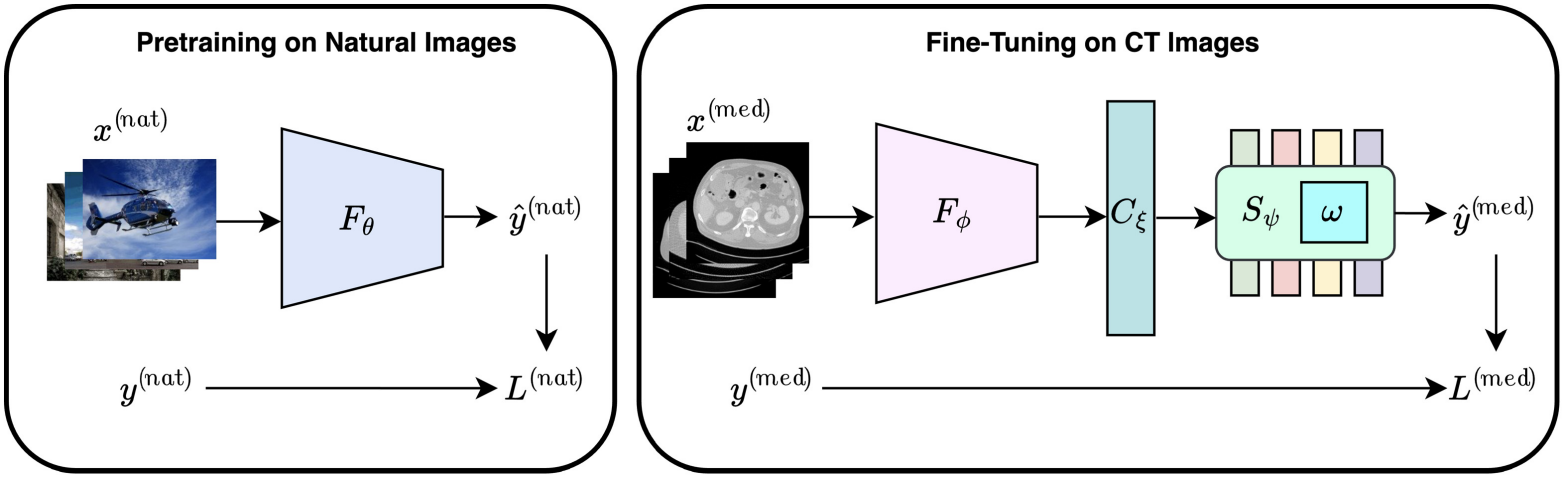
Schematic diagram of the proposed TFKT V2 method. During the first phase, the EfficientNetV2L backbone network ($F_{\theta }$) is trained to predict MOS scores from input natural images. In the second phase, low-dose CT images are fed to the pre-trained EfficientNet to extract local features by transferring knowledge from natural image pre-training. A bridge connection ($C_{\xi }$) is introduced that connects and feeds extracted local features from EfficientNet ($F_{\phi }$) to our modified Swin Transformer (Swin-KAT) ($S_{\psi }$) to exploit local–global features and predict the final IQA scores. For inference, the model takes a CT image as input and predicts the corresponding IQA score.

**Fig. 2:**
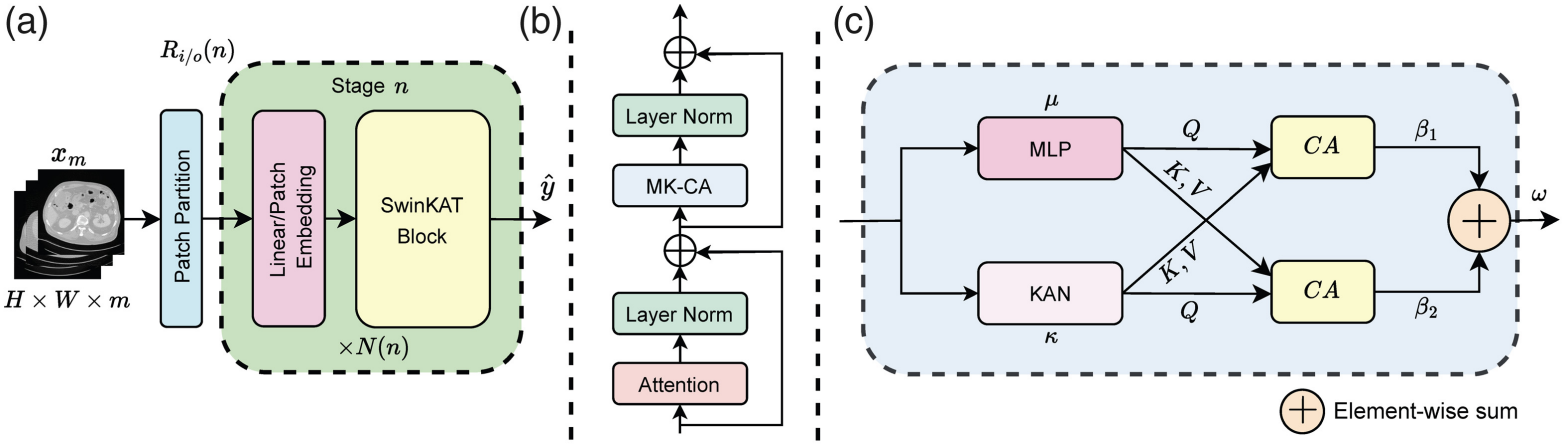
Proposed Swin-KAT: Swin-KAT blocks modify the Swin Transformer to help capture hierarchical visual features more efficiently. MLP-KAN combined via dual cross-attention paths (MK-CA) is embedded in each of the Swin-KAT blocks. The attentions calculated from each path are summed up to generate the output of the block. (a) Swin-KAT overview. (b) Swin-KAT block. (c) MK-CA.

To formulate the problem, we suppose $D_{\mathrm{nat}}=\{X^{\left(\mathrm{nat}\right)},Y^{\left(\mathrm{nat}\right)}\}$ be a natural image dataset sampled i.i.d. from $p(X^{\left(\mathrm{nat}\right)},Y^{\left(\mathrm{nat}\right)})$, where $X_{\mathrm{nat}}=\{x_{1}^{\left(\mathrm{nat}\right)},x_{2}^{\left(\mathrm{nat}\right)},\ldots ,x_{n1}^{\left(\mathrm{nat}\right)}\}$ denotes the set of given natural images of different qualities (acquired with distortions) and $Y_{\mathrm{nat}}=\{y_{1}^{\left(\mathrm{nat}\right)},y_{2}^{\left(\mathrm{nat}\right)},\ldots ,y_{n1}^{\left(\mathrm{nat}\right)}\}$ denotes the set of corresponding MOS scores. Similarly, we have a much smaller medical (CT) image dataset $D_{\mathrm{med}}=\{X^{\left(\mathrm{med}\right)},Y^{\left(\mathrm{med}\right)}\}$ sampled i.i.d. from $p(X^{\left(\mathrm{med}\right)},Y^{\left(\mathrm{med}\right)})$, where $X^{\left(\mathrm{med}\right)}=\{x_{1}^{\left(\mathrm{med}\right)},x_{2}^{\left(\mathrm{med}\right)},\ldots ,x_{n2}^{\left(\mathrm{med}\right)}\}$ denotes the set of given noisy images (acquired at a lower dose and different artifacts) and $Y_{\mathrm{nat}}=\{y_{1}^{\left(\mathrm{med}\right)},y_{2}^{\left(\mathrm{med}\right)},\ldots ,y_{n2}^{\left(\mathrm{med}\right)}\}$ denotes the set of corresponding IQA scores. We assume that the $D_{\mathrm{med}}$ is much smaller compared with $D_{\mathrm{nat}}$, i.e., n2≪n1. In the proposed TFKT V2, we utilize a CNN backbone as EfficientNet for pre-training on $D_{\mathrm{nat}}$ and then combine it with a modified transformer backbone as Swin Transformer (Swin-KAT) through a connector during fine-tuning on $D_{\mathrm{med}}$ (Fig. 1). Our goal is to train the deep learning model that can estimate $y_{i}^{\left(\mathrm{nat}\right)}$ given $x_{i}^{\left(\mathrm{nat}\right)}$ subsequently $y_{i}^{\left(\mathrm{med}\right)}$ given $x_{i}^{\left(\mathrm{med}\right)}$. Moreover, we have made modifications to the transformer model to enhance its performance (Fig. 2).

### 3.4 Swin-KAT

Figure 2(a) illustrates the proposed Swin-KAT model. Swin-KAT based on Swin Transformer^38^ consists of hierarchical feature maps with shifted windows and transformer blocks, where N denotes the number of repetitions of the procedure and n is the number of stages adopted by the Swin Transformer^37^ architecture. $R_{i/o}(n)$ is denoted by input/output resolution where $R_{i/o}(n)=(\frac{H}{d_{n}},\frac{W}{d_{n}},C_{n})$ and n∈{1,2,3,4}. Here, $d_{n}$ is the downsampling factor, and $C_{n}$ is the channel multiplier based on the original number of channels. As illustrated in Fig. 2(b), our proposed MLP-KAN cross attention (MK-CA) is embedded within the Swin-KAT block. We use spline-based univariate function KANs,^23^ which leads to better neural scaling and improved generalization in regression tasks compared with traditional MLPs in particular cases.

#### 3.4.1 MLP-KAN cross attention

In our proposed MK-CA block Fig. 2(c), we incorporate MLP and KAN in parallel, employing dual path cross-attentions

$$\beta_{1}=\text{CrossAttention}(Q_{\mu },K_{\kappa_{\eta }},V_{\kappa_{\eta }}),$$ (1)

$$\beta_{2}=\text{CrossAttention}(Q_{\kappa_{\eta }},K_{\mu },V_{\mu }),$$ (2)

where β denotes the output from the cross-attention mechanism, and Q, K, and V represent the query, key, and value matrices, respectively. μ corresponds to the MLP, whereas κ refers to the KAN block, with η representing the number of heads in KAN. The number of heads conveys the attention mechanism in κ and divides the input into η parallel heads, each attending to different parts of the input features. The resulting outputs $\beta_{1}$ and $\beta_{2}$ are then combined using element-wise summation as

$$\omega =\beta_{1}+\beta_{2}.$$ (3)

Therefore, the final output of MK-CA is computed as ω, combining information from both cross-attention pathways.

#### 3.4.2 Natural image (MOS) pre-training

During the pre-training phase, our proposed TFKT V2 model is trained on predicting the MOS scores from input natural images, as shown in Fig. 1. Leveraging a CNN backbone, TFKT V2 performs a regression task to predict a real-valued image quality score within $\left\{s_{\min}^{\left(\mathrm{nat}\right)},s_{\max}^{\left(\mathrm{nat}\right)}\right\}$ from any given input $x_{i}^{\left(\mathrm{nat}\right)}$. Considering the smaller model and its faster training, we use ImageNet pre-trained EfficientNetV2L^36^ as the CNN backbone architecture (F). The CNN regressor $\left(F_{\theta }\right)$ (θ denotes the set of parameters) is trained on optimizing an L2 loss calculated from the MOS reference $y_{i}^{\left(\mathrm{nat}\right)}$. The predicted score is obtained as

y^i(nat)=Fθ(xi(nat));i=1,2,…,M, (4)

where M is the minibatch size. Then, the loss is calculated per minibatch as

Lnat=∑iM‖yi(nat)−y^i(nat)‖2. (5)

#### 3.4.3 CT image (LDCTIQA) fine-tuning

Once the model $F_{\theta }$ is trained on predicting MOS scores of natural images from the pre-training phase, we utilize the learned model $F_{\theta }$ and feed the model with CT images. The final output layer from MOS pre-training is dropped, and $F_{\phi }$ is used for feature extraction, where ϕ⊂θ. During the fine-tuning phase, we also add a transformer backbone to exploit both local and global features in medical images. Specifically, we take the ImageNet pre-trained Swin-KAT S, which is a modified version of SwinV2B^38^ as an effective architecture for general purpose vision tasks. To avoid any negative transfer of knowledge from natural image pre-training, TFKT V2 makes use of an extended modified model S during the fine-tuning stage. This enables the model to exploit the task-specific knowledge transferred from the natural image pre-training and, at the same time, draw the domain-specific information from the CT images themselves.

Therefore, our final extended TFKT model (H) comprises the pre-trained F, transformer-based S, and a small connector module C to bridge CNN and transformer networks. Therefore

$$H_{\Omega }=\{F_{\phi }\cup C_{\xi }\cup S_{\psi }\},$$ (6)

where ξ and ψ denote the parameters in C and S networks, respectively, and Ω denotes the parameters in the whole model H.

The connector C is a small module that bridges between F and S so that local and global features can be smoothly exploited for final IQA prediction. C comprises a fully connected layer to map extracted features from $F_{\phi }$ at 1280 to 4096, which is followed by a ReLU activation and reshaped to 64×64 for input to Swin-KAT ($S_{\psi }$).

Now, the hybrid model $H_{\Omega }$ as in TFKT V2 is trained on predicting the perceptual IQA scores in the range $\left\{s_{\min}^{\left(\mathrm{med}\right)},s_{\max}^{\left(\mathrm{med}\right)}\right\}$ for any given CT image input $x_{i}^{\left(\mathrm{med}\right)}$. Similar to pre-training, the $H_{\Omega }$ regressor is also trained on optimizing an L2 loss calculated from the CT IQA reference $y_{i}^{\left(\mathrm{med}\right)}$. The predicted score ${\hat{y}}_{i}^{\left(\mathrm{med}\right)}$ is obtained, and loss is calculated

y^i(med)=Fθ(xi(med));i=1,2,…,M, (7)

Lmed=∑iM‖yi(med)−y^i(med)‖2. (8)

Unlike many other natural-to-medical data transfer learning approaches, TFKT V2 does not require any modifications to hyperparameter choices and the loss function for training on medical images.

#### 3.4.4 Training objectives

Algorithm 1 outlines our two-phase training strategy, which begins with pre-training on natural images to learn robust feature representations. This is followed by fine-tuning the CT image dataset, allowing the model to adapt to the domain-specific features of medical imaging. This approach efficiently transfers knowledge from natural images to CT images and enables the hybrid model to achieve accurate image quality predictions even with limited CT data.

**Algorithm 1:** Two-phase CT image quality assessment.

1: **Input:** Natural image dataset $D_{\mathrm{nat}}=\{X^{\left(\mathrm{nat}\right)},Y^{\left(\mathrm{nat}\right)}\}$, CT image dataset $D_{\mathrm{med}}=\{X^{\left(\mathrm{med}\right)},Y^{\left(\mathrm{med}\right)}\}$, epochs $T_{\text{pretrain}}$ and $T_{\text{fine-tune}}$, optimizer OPT.
2: **Output:** Trained hybrid model $H_{\Omega }$.
3:
4: **Phase 1: Pre-training EfficientNet** $F_{\phi }$
5: Initialize EfficientNet $F_{\phi }$ and optimizer OPT for $F_{\phi }$.
6: **for** t=1 to $T_{\text{pretrain}}$ **do**
7: **for** each minibatch $\{X_{b}^{\left(\mathrm{nat}\right)},Y_{b}^{\left(\mathrm{nat}\right)}\}\in D_{\mathrm{nat}}$ **do**
8: Predict: ${\hat{Y}}_{b}^{\left(\mathrm{nat}\right)}=F_{\phi }(X_{b}^{\left(\mathrm{nat}\right)})$.
9: Compute loss: Lnat=∑i=1M‖yi(nat)−y^i(nat)‖2.
10: Update $F_{\phi }$ using OPT to minimize $L_{\mathrm{nat}}$.
11: **end for**
12: **end for**
13: Save pretrained weights $\phi_{\text{pretrained}}$.
14:
15: **Phase 2: Fine-tuning hybrid model** $H_{\Omega }$
16: Load pretrained weights $\phi_{\text{pretrained}}$ into $F_{\phi }$.
17: Define hybrid model: $H_{\Omega }=\{F_{\phi }\cup C_{\xi }\cup S_{\psi }\}$.
18: Initialize connector $C_{\xi }$, Swin Transformer $S_{\psi }$, and optimizer OPT for $H_{\Omega }$.
19: **for** t=1 to $T_{\text{fine-tune}}$ **do**
20: **for** each minibatch $\{X_{b}^{\left(\mathrm{med}\right)},Y_{b}^{\left(\mathrm{med}\right)}\}\in D_{\mathrm{med}}$ **do**
21: Extract features: $Z_{b}^{\left(\mathrm{med}\right)}=F_{\phi }(X_{b}^{\left(\mathrm{med}\right)})$.
22: Predict: ${\hat{Y}}_{b}^{\left(\mathrm{med}\right)}=S_{\psi }(C_{\xi }(Z_{b}^{\left(\mathrm{med}\right)}))$.
23: Compute loss: Lmed=∑i=1M‖yi(med)−y^i(med)‖2.
24: Update $H_{\Omega }$ parameters using OPT to minimize $L_{\mathrm{med}}$.
25: **end for**
26: **end for**
27:
28: **Return:** Trained hybrid model $H_{\Omega }$.

#### 3.4.5 Model inference

**Fig. 3:**
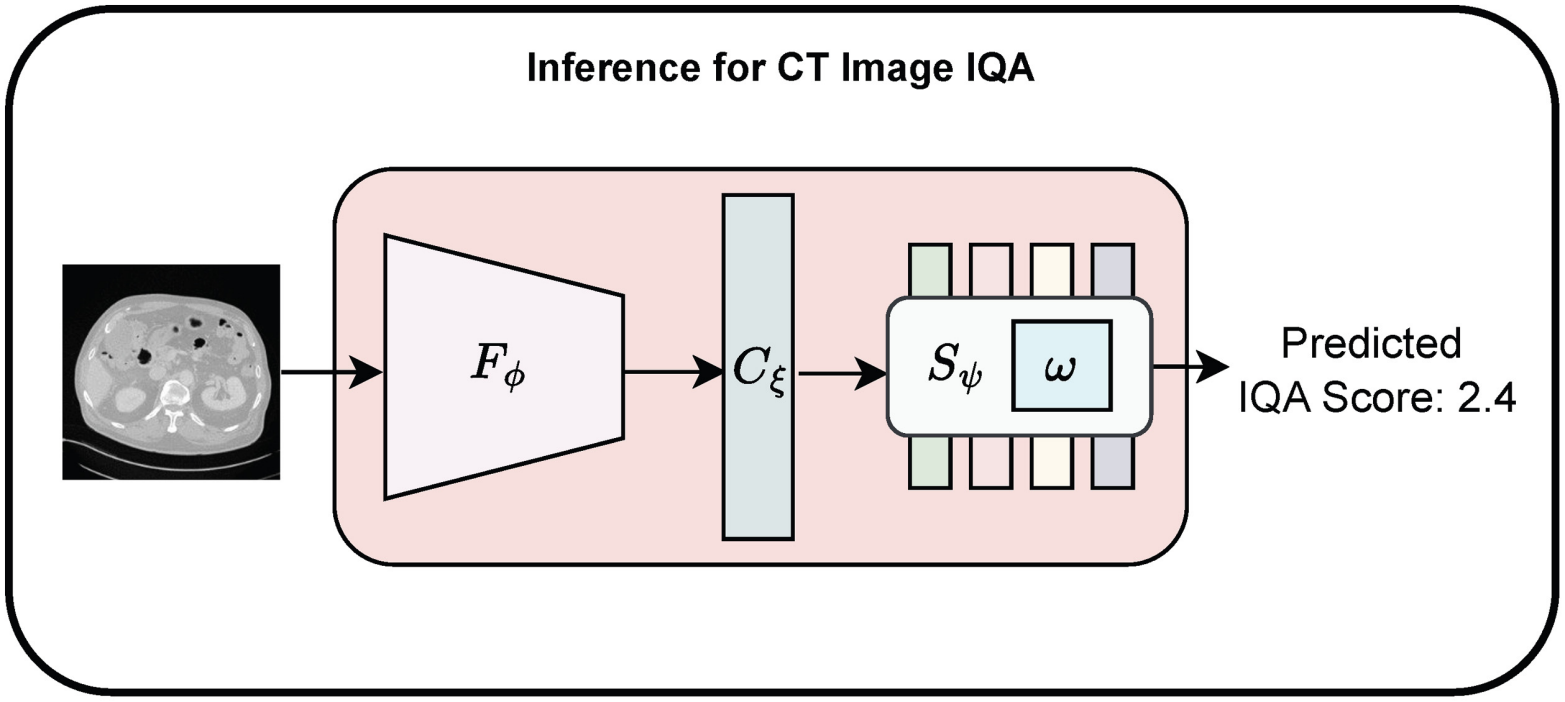
TFKT V2 inference for predicting IQA for an input CT image.

For model inference, the fine-tuned hybrid model from the second phase shown in Algorithm 1 is utilized. The pre-trained EfficientNet extracts CT image features which are processed by the connector module and modified Swin-KAT to predict the final IQA score (Fig. 3). Note that once TFKT V2 is fully trained, the natural image data are no longer required at inference time. The model relies solely on the features learned during fine-tuning with the CT image dataset. With a single forward pass of the model, the IQA score for an input CT image can be predicted.

## 4 Experiments and Results

### 4.1 Datasets

#### 4.1.1 LDCTIQA

**Fig. 4:**
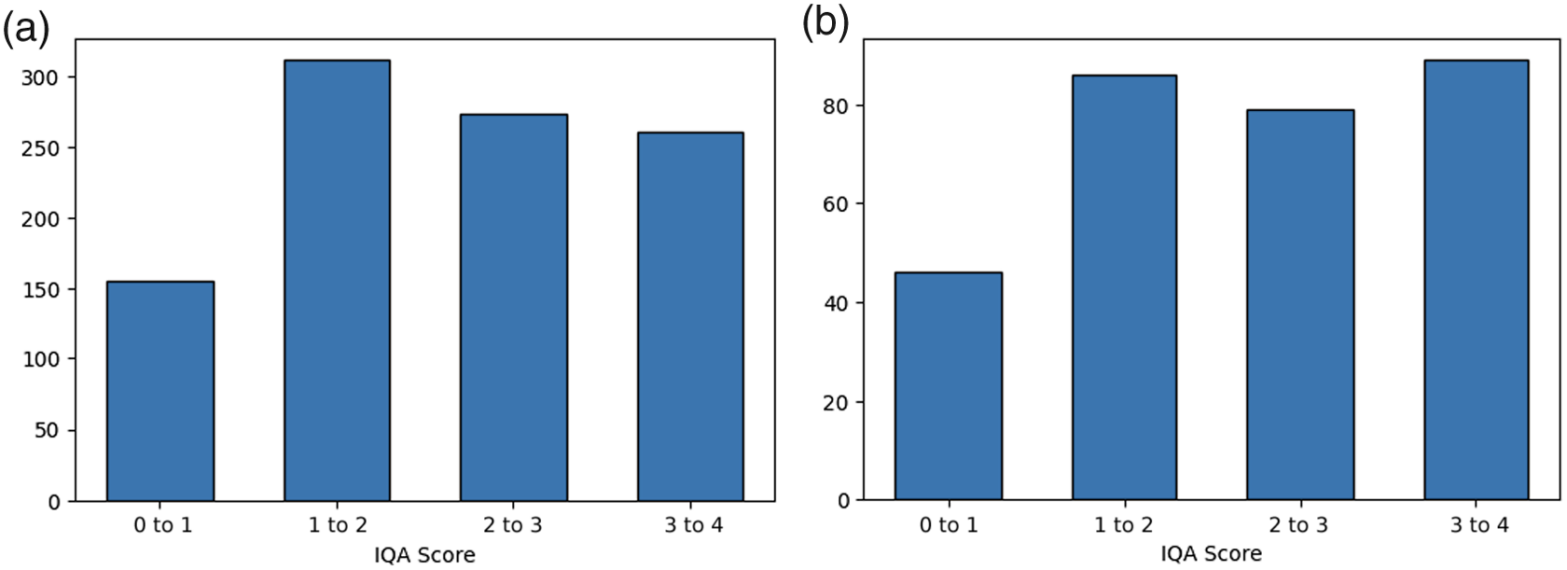
IQA score distributions across different quality levels (0 to 4). (a) Training set. (b) Test set.

We train and evaluate our method using the low-dose CT perceptual image quality assessment challenge 2023 (LDCTIQA) dataset. The dataset includes both training and testing sets. The training set consists of 1000 axial CT slices, whereas the testing set contains 300 axial CT slices with varying levels of distortion. Image quality in the training and testing sets was assessed by five expert radiologists and six radiologists, respectively. Quality scores range from 0 to 4, where a score of 4 denotes excellent quality (with highly visible anatomical structures), and a score of 0 indicates poor quality (with desired features not visible) (Table 1). The final image quality score for each image is determined by averaging the scores assigned by radiologists, resulting in an IQA score similar to the MOS score in KADID-10k. All images have a resolution of 512×512  pixels. In addition, the distribution of IQA scores across different quality levels for both the training and test sets is shown in Fig. 4.

#### 4.1.2 KADID-10k

**Fig. 5:**
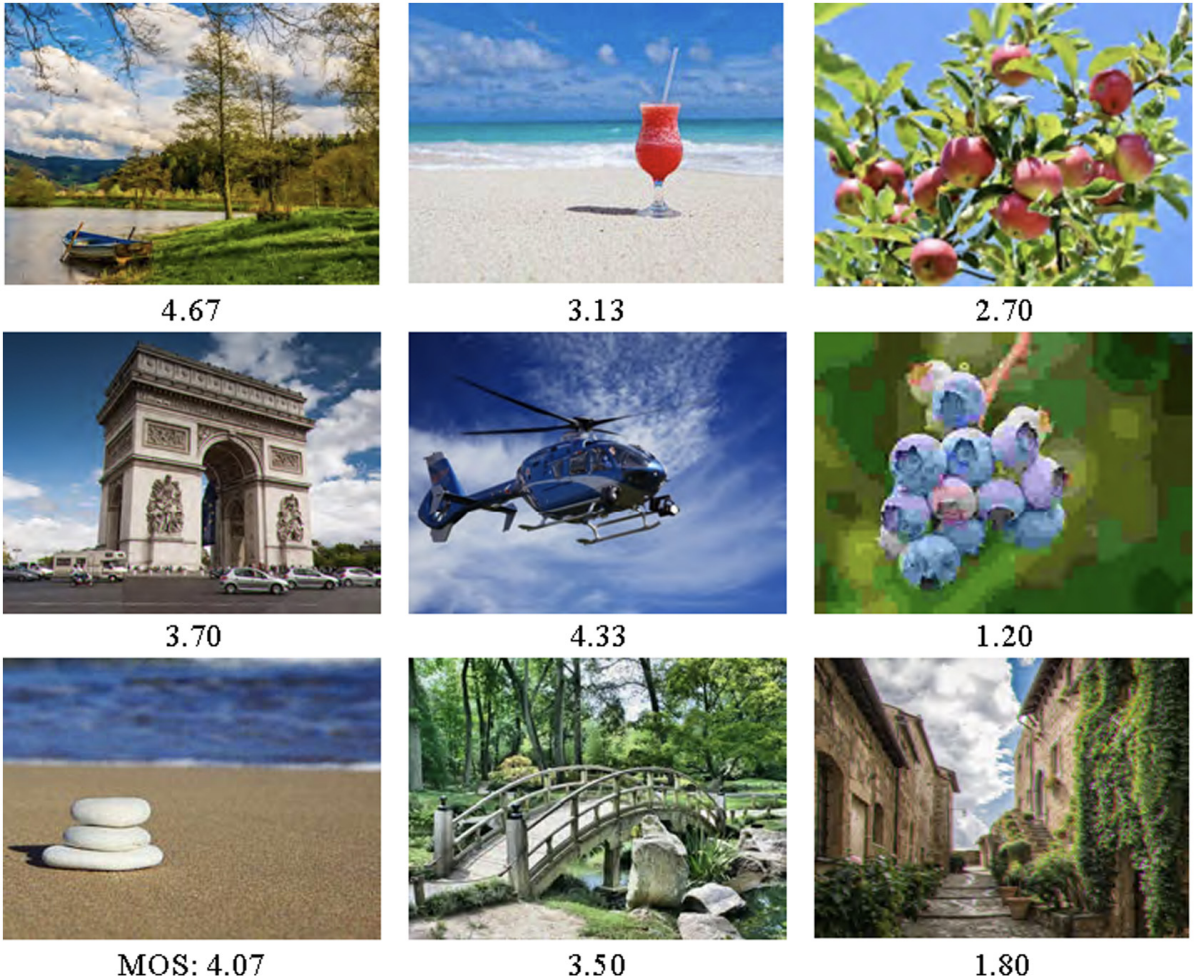
Sample images from the KADID-10k dataset with varying image quality scores (MOS: 1 for bad and 5 for excellent).

We leverage natural images from the KADID-10k^10^, which contains 81 pristine or original images. Figure 5 shows representative samples from the KADID-10k dataset. The quality of each pristine image was artificially degraded using 25 distortion types (e.g., blurs, color distortions, noise, brightness, and contrast adjustments) applied at five different severity levels, resulting in a total of 10,125 images in the dataset. The KADID-10k dataset includes a MOS score for each distorted image. The MOS ranges among predefined values, reflecting perceived image quality based on human assessments. This dataset serves as a valuable benchmark for evaluating image quality prediction models.

### 4.2 Implementation Details

#### 4.2.1 Experimental setup

To align with the CT IQA scale, we adjust the MOS scores of the natural images. The input images are min–max normalized and resized to 512×512  pixels if their original sizes are different. We evaluated various established and state-of-the-art architectures, demonstrating generalizability across architectural choices. In all cases, the input layer is customized to facilitate single-channel gray-scale inputs. The final output layer is also adjusted for the models to predict a single regression value within the IQA range. The models were implemented in Python using PyTorch, running on an Intel(R) Xeon(R) W7-2475X processor (2600 MHz) with dual NVIDIA A4000X2 GPUs (32 GB). We employed the Adam optimizer with an initial learning rate of $1\times 10^{-4}$, using a cosine annealing-based learning rate scheduler. For consistency, all models were trained for 100 epochs with a mini-batch size of 4.

Our proposed TFKT V2 model was compared against several baseline and state-of-the-art IQA models, including deep bilinear convolutional neural network (DBCNN),^39^ MD-IQA,^27^ multi-dimension attention network (MANIQA),^28^ attention-based hybrid IQA model (AHIQ),^29^ transformers, relative ranking, and self consistency, (TReS),^40^ EfficientNetV2L,^36^ quality-aware pre-trained (QPT),^30^ distortion-based blind IQA (DB-IQA),^5^ and self-supervised IQA (SSIQA).^2^ We also experimented with different TFKT model variants to determine the optimal settings.

#### 4.2.2 Training and evaluation

Model performance was evaluated using fivefold cross-validation with 200 CT images from the training set of the LDCTIQA dataset per fold. We conducted holdout validation using two experimental setups. For the first setting (setting A), we reserved 200 images as the test set and used the remaining 800 images from the LDCTIQA training set for training. This setup allows a fair comparison with state-of-the-art models, which were evaluated similarly without using the official test set. For the second setting (setting B), we trained the model with 900 images from the training set and used 100 images for validation. The evaluation was carried out on the official LDCTIQA test set containing 300 images. All validation results reported in the paper are based on setting A while testing results are derived from setting B.

### 4.3 Evaluation Measures

Following,^8^ we evaluate the model performance using both linear and non-linear correlation coefficients for robust evaluation of the model performance: Pearson’s linear correlation coefficient (r), Spearman’s rank correlation coefficient (ρ), and Kendall rank correlation coefficient (τ). From the reference and predicted IQA scores, the three correlation coefficients can be calculated as

r=∑i=1n(yi−y¯)(y^i−y^¯)∑i=1n(yi−y¯)2∑i=1n(y^i−y¯)2, (9)

where $\overset{¯}{y}$ and y^¯ denote the mean of y and $\hat{y}$, respectively.

$$\rho =1-\frac{6\overset{n}{\underset{i=1}{\sum }}d_{i}^{2}}{n(n^{2}-1)},$$ (10)

where $d_{i}={\hat{y}}_{i}-y_{i}$ is the difference between the i’th image’s ranks in the reference and predicted assessments

$$\tau =\frac{P-Q}{\sqrt{\left(P+Q+y_{0})(P+Q+{\hat{y}}_{0}\right)}},$$ (11)

where P is the number of concordant pairs, Q is the number of discordant pairs, $y_{0}$ is the number of pairs tied only on the radiologists’ IQA scores, and ${\hat{y}}_{0}$ is the number of pairs tied only on the model-predicted scores.

The overall model performances for image quality assessment are evaluated by aggregating the values of all three correlation coefficients from Eqs. (9)–(11)

s=|r|+|ρ|+|τ|. (12)

### 4.4 Results and Discussion

Table 2 reports the quantitative performances of our proposed TFKT V2 method using the three individual correlation coefficient metrics: r, ρ, and τ and their sum for the overall performance s. Comparison against state-of-the-art and existing CNN and transformer-based methods reveals the superiority of our TFKT V2 method in effectively quantifying the quality of lower-dose CT images and outperforming all the compared methods.

**Table 2:** Quantitative comparison against baseline and state-of-the-art methods in setting A. We report the individual correlation coefficients as well as the overall (sum) IQA scores. The best and second-best results are bolded and italicized, respectively.

Methods	Performance metrics			
	r	ρ	τ	s
DBCNN^39^	0.9714	0.9734	0.8808	2.8255
MD-IQA^27^	0.9771	0.9793	0.9106	2.8670
MANIQA^28^	0.9768	0.9786	0.8891	2.8445
AHIQ^29^	0.9762	0.9746	0.8810	2.8317
TReS^40^	0.9755	0.9745	0.8786	2.8286
EfficientNetV2L^36^	0.9569	0.9741	0.8772	2.8082
QPT^30^	0.9743	0.9732	0.8797	2.8272
SSIQA^2^	0.9784	0.9767	0.8905	2.8456
D-BIQA^5^	0.9814	*0.9816*	*0.9122*	*2.8753*
Ensemble(EffcientNet+SwinV2B)	0.9787	0.9790	0.8932	2.8509
TFKT–frozen F^11^	0.9724	0.9757	0.8852	2.8332
TFKT^11^	*0.9828*	0.9801	0.9005	2.8634
TFKT V2	**0.9858**	**0.9861**	**0.9171**	**2.8890**

In terms of r and ρ, the TFKT^11^ model and D-BIQA^5^ have shown strong performance as yet, whereas TFKT V2 surpasses it, demonstrating the effectiveness of integrating the combined MLP and KAN into the Swin Transformer while leveraging EfficientNet pre-training. Also, compared with MD-IQA^27^ and D-BIQA,^5^ TFKT V2 achieves superior performance in terms of τ. The performance of MD-IQA is limited to unstable training and quality of labels, which may lead to poor out-of-domain generalization. Also, MD-IQA is reliant on a large amount of unlabeled data, which can be challenging to obtain in some settings. On the other hand, D-BIQA generates a reference image and then compares the input image against it to perform an image quality assessment. This approach makes it not a true no-reference IQA method. The superiority of TFKT V2 over the ensemble (averaging individual predictions) of two backbone networks (EfficientNet and Swin Transformer) further justifies the proposed knowledge transfer and the addition of the bridge module C among them. We also experiment by keeping the pre-trained weights in F frozen during the fine-tuning stage, and the results suggest the usefulness of fine-tuning the full H model. We performed the Wilcoxon signed-rank test by taking IQA residuals (errors in predictions for the test samples) for each of the TFKT and TFKT V2 models. The tests confirmed that our TFKT V2 is significantly better than TFKT (p-value<0.001).

In a fivefold cross-validation setting with the training set, we perform the direct prediction of the IQA score from CT images using different backbone architectures. As shown in Table 3, EfficientNetV2L consistently performs better than other CNN-based models in terms of the correlation coefficient metrics and their sum. Also, the Swin Transformer performs better than other transformer-based models. This justifies the selection of backbone architecture as EfficientNetV2L and Swin Transformer for our task-focused transfer learning approach. Furthermore, we performed the Wilcoxon signed-rank test by taking IQA residuals (errors in predictions for the test samples) for each of the EfficientNet, ResNet, ViT, and SwinV2B models. The tests confirmed that EfficientNet and SwinV2B are significantly better than their CNN and transformer counterparts, respectively (p-value<0.001).

**Table 3:** Quantitative comparison of the IQA score prediction using different backbone architectures in a fivefold cross-validation setting. We report the mean and variance IQA scores for the individual and overall correlation coefficient metrics. The best and second-best results are bolded and italicized, respectively.

Methods	Performance metrics			
	r	ρ	τ	s
ResNet-18	0.9782 ± 0.0029	0.9586 ± 0.0083	**0.8997 ± 0.0138**	*2.8364 ± 0.0232*
ResNet-50	*0.9784 ± 0.0034*	0.9580 ± 0.0131	0.8934 ± 0.0275	2.8298 ± 0.0423
EfficientNetB4	0.9757 ± 0.0037	*0.9748 ± 0.0042*	0.8816 ± 0.0090	2.8322 ± 0.0169
EfficientNetV2L	**0.9807 ± 0.0025**	**0.9809 ± 0.0025**	*0.8982 ± 0.0068*	**2.8598 ± 0.0116**
ConvNeXt	0.9748 ± 0.0032	0.9733 ± 0.0037	0.8771 ± 0.0106	2.8252 ± 0.0171
ViT	0.8879 ± 0.0129	0.8863 ± 0.0186	0.7219 ± 0.0239	2.4961 ± 0.0548
SwinV2B	0.9754 ± 0.0067	0.9745 ± 0.0077	0.8839 ± 0.0177	2.8338 ± 0.0321

Furthermore, we conducted experiments using only the Swin Transformer, Swin-KAN, which replaces the MLP with KAN in the Swin Transformer, and Swin-KAT, which is the combination of MLP and KAN with a cross-attention mechanism. Table 4 shows that the combined MLP and KAN perform better than other Swin Transformer approaches in both setting A and setting B. We also performed statistical tests to compare Swin-KAT against Swin-KAN and Swin Transformer in settings A and B. We performed the Wilcoxon signed-rank test by taking IQA residuals (errors in predictions for the test samples) for each of the three models. The tests confirmed that our Swin-KAT is significantly better than the Swin-KAN and Swin Transformer models across both settings (p-value<0.001).

**Table 4:** Quantitative comparison of Swin Transformer, Swin-KAN, and Swin-KAT for CT IQA, highlighting Swin-KAT as the most suitable method in terms of settings A and B. The best results are bolded.

Methods	Setting A				Setting B			
	r	ρ	τ	s	r	ρ	τ	s
Swin Transformer	0.9796	0.9810	0.8923	2.8529	0.9331	0.9331	0.7854	2.6516
Swin-KAN	0.9833	0.9822	0.9031	2.8685	0.9373	0.9382	0.7954	2.6709
Swin-KAT	**0.9831**	**0.9825**	**0.9031**	**2.8687**	**0.9454**	**0.9389**	**0.7967**	**2.6811**

To verify the need for EfficientNet in the TFKT framework, we experiment with the transformer-only models and report their performances in Table 5. The three variants of Swin Transformer, namely, Swin Transformer, Swin-KAN, and Swin-KAT, were pre-trained on the KADID natural image dataset followed by fine-tuning on the LDCTIQA dataset. We find Swin-KAT as the superior among the three variants consistent with the results from no pre-training (Table 4). Although the pre-training helps improve the performance over its no pre-training counterpart, the poorer scores across all the metrics compared with the TFKT V2 framework justify the incorporation of EfficientNet in both phases. We performed the Wilcoxon signed-rank test by taking IQA residuals (errors in predictions for the test samples) for each of the three models and obtained similar results as in Table 4 across both settings (p-value<0.001).

**Table 5:** Quantitative evaluation of the validation and testing datasets. Pre-training was conducted with the KADID10K dataset and fine-tuning with the LDCTIQA dataset. The best results are bolded.

Methods	Setting A				Setting B			
	r	ρ	τ	s	r	ρ	τ	s
Swin Transformer	0.9878	0.9799	0.8935	2.8612	0.9380	0.9329	0.7872	2.6581
Swin-KAN	0.9876	0.9821	0.9056	2.8753	0.9396	0.9388	0.7963	2.6747
Swin-KAT	**0.9868**	**0.9833**	**0.9061**	**2.8762**	**0.9467**	**0.9414**	**0.7998**	**2.6879**

**Fig. 6:**
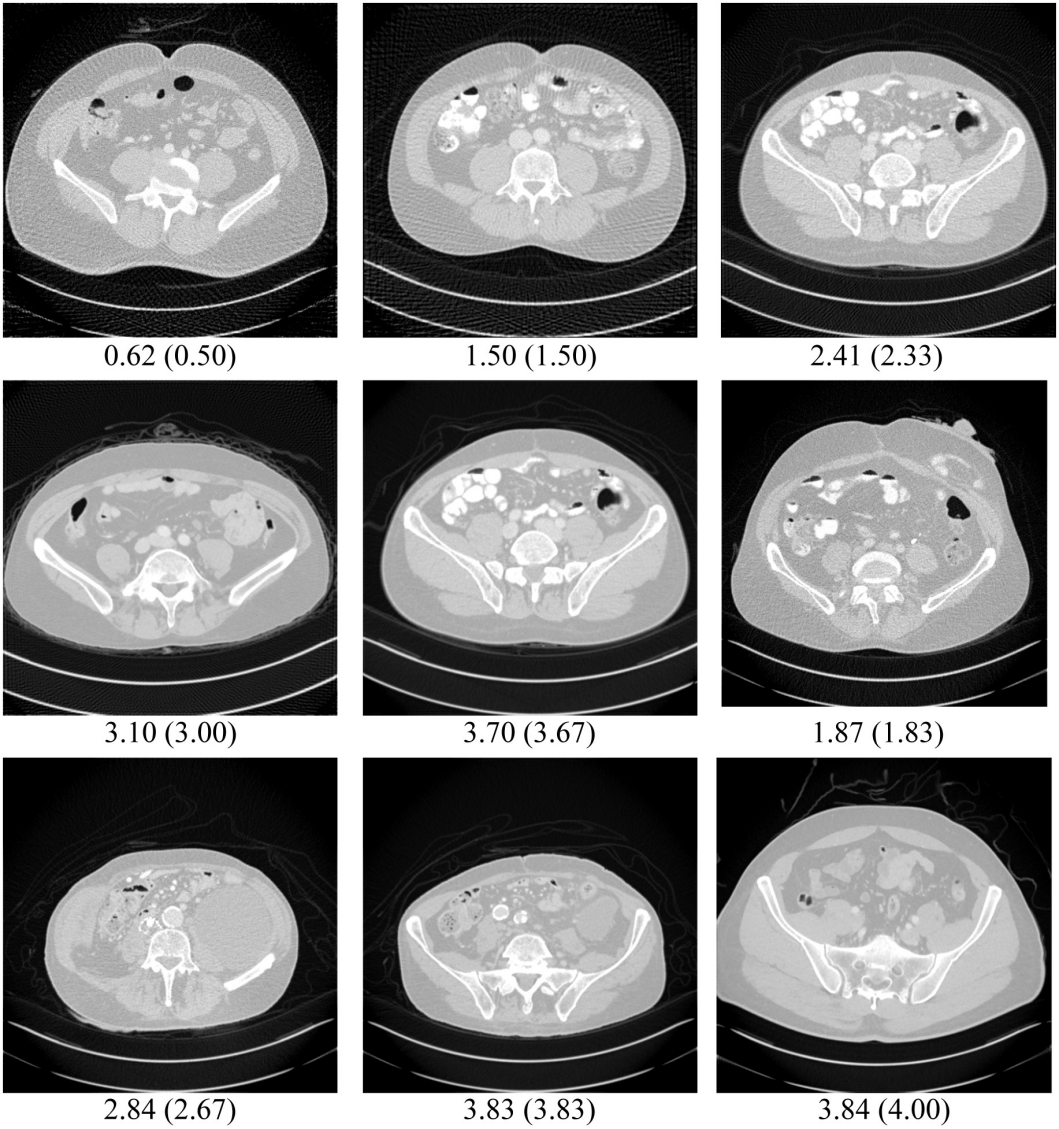
Effectiveness of our TFKT V2 model in accurately assessing the quality of abdominal CT images across diverse IQA scores. Model predictions are in good agreement with the (actual) scores (window width: 1300; level: −800).

Figure 6 visualizes some sample CT images of varying IQA scores—different levels of noise and artifacts from the LDCTIQA test set. Comparing the model-predicted IQA scores, we observe good agreement with the reference scores. This showcases the robustness of TFKT V2 in effectively quantifying diverse-quality CT images.

**Fig. 7:**
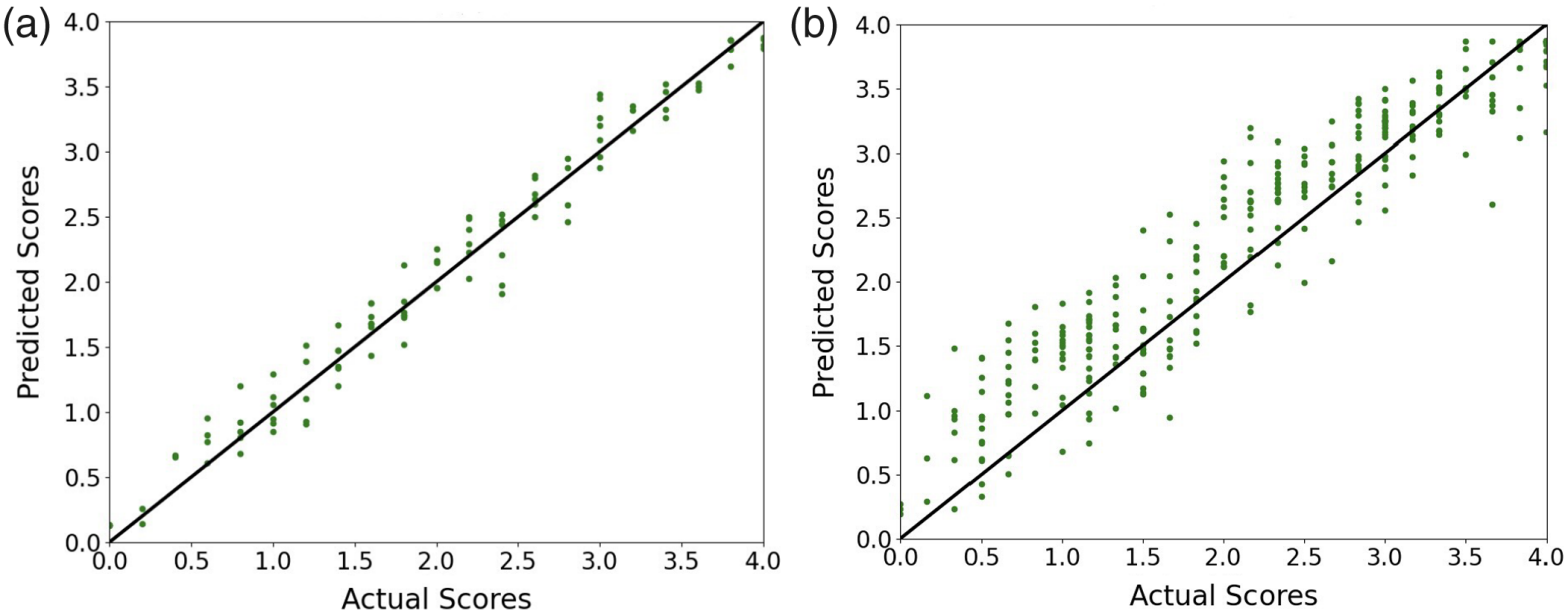
Scatter plots demonstrating the relationship between actual and predicted scores in both settings. (a) Setting A. (b) Setting B.

We also provide a visual representation of the coefficient of determination ($R^{2}$ score) to measure how well the predicted values match the actual ground truth values. This indicates how much of the variance in the actual values can be explained by our model’s predictions. Figure 7 depicts the relationship between actual and predicted scores. Figures 7(a) and 7(b) highlight the performance with settings A and B, respectively. The higher $R^{2}$ score (close to 1) demonstrates that our model’s predictions are very close to the actual values and that it explains 97% and 85% of the variance in the data for settings A and B, respectively. Moreover, setting A corresponds to a correlation of r≈0.985, and setting B corresponds to r≈0.927, indicating consistently strong positive correlations in the two settings.

**Fig. 8:**
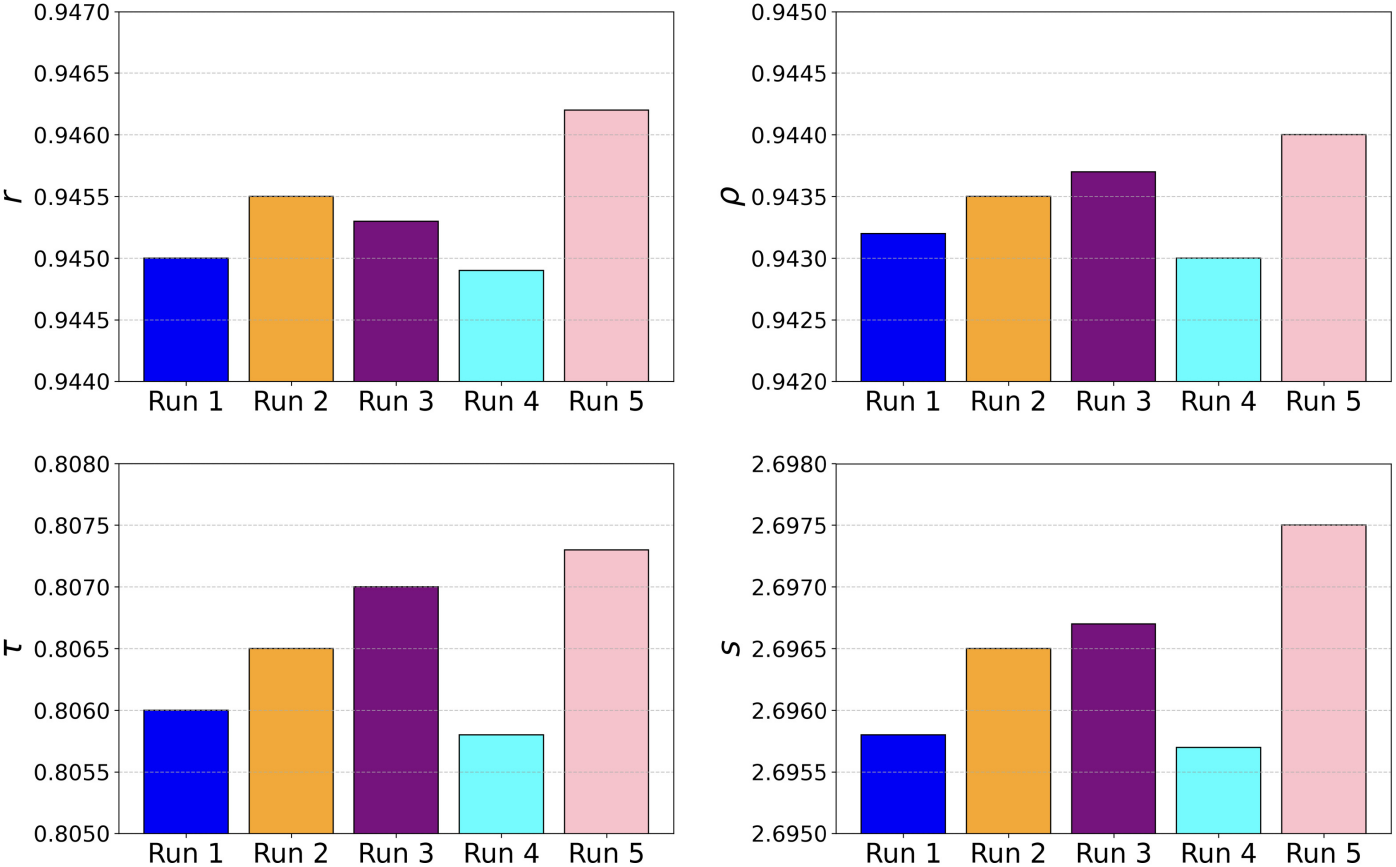
Consistency of the TFKT model performance across five runs for the four evaluation metrics (r, ρ, τ, and s).

We ran our TFKT V2 model five times and evaluated by calculating the metrics in setting B. As shown in Fig. 8, the model performs consistently across the five runs and for four evaluation metrics: r, ρ, τ, and s. Each of the evaluation metrics shown independently highlights variability across the runs. The mean overall score in all runs is 2.6964 with a very low variance of $4.30\times 10^{-7}$. This indicates the high consistency of our proposed TFKT V2 model. This is further confirmed by performing the Kruskal–Wallis H-test,^41^ comparing the predictions and the IQA residuals of the five model runs. We found no significant differences (p-value>0.05) for run 5 compared with the other four. This analysis provides insights into the model’s robustness and stability over repeated trials.

#### 4.4.1 Ablation study

We experiment with different possible operations that combine MLP and KAN layers in our proposed Swin-KAT architecture. As reported in Table 6, the experimented operations include only MLP, only KAN, average, concatenation, summation, MLP cross-attention (Q=μ, K=V=κ), KAN cross-attention (Q=κ, K=V=μ), and our proposed approach based on MK-CA as ω. The model performs inferiorly using only MLP or only KAN compared with the approaches that combine them. This highlights the importance of adopting a combined MLP-KAN strategy to achieve optimal results. The experiment provided critical insights that informed the selection of our final approach, following setting B for evaluation and using only the Swin Transformer for the experiment. This is further confirmed by performing Wilcoxon signed-rank tests for μ−κ with SUM and ω with CA model. The best model is based on the optimal ω model as p-value<0.001. We also experimented with different numbers of KAN heads (Table 7) to determine the optimal one. We find that the optimal number of heads is 16. This is further confirmed by performing a Wilcoxon signed-rank test similar to earlier experiments comparing models with 8 and 16 heads, where the best performance was achieved with 16 heads (p-value<0.001).

**Table 6:** Ablation study (setting B) based on the quantitative evaluation of different combinations of MLP and KAN with varying operations demonstrates the superiority of the proposed MK-CA (ω). The best results are bolded.

Methods	Operation	r	ρ	τ	s
μ only	—	0.9331	0.9331	0.7854	2.6516
κ only	—	0.9373	0.9382	0.7954	2.6709
μ-κ	Average	0.9405	0.9375	0.7938	2.6718
μ-κ	Concat	0.9391	0.9367	0.7902	2.6659
μ-κ	Sum	0.9436	0.9380	0.7921	2.6738
μ-κ	CA	0.9352	0.9321	0.7855	2.6529
μ-κ	CA	0.9368	0.9298	0.7793	2.6460
ω	CA	**0.9454**	**0.9389**	**0.7967**	**2.6811**

**Table 7:** Ablation study to select the number of KAN heads (η) in Swin-KAT using setting B. The best results are bolded.

η	r	ρ	τ	s
8	0.9443	0.9363	0.7925	2.6731
16	**0.9454**	**0.9389**	**0.7967**	**2.6811**
32	0.9445	0.9370	0.7915	2.6730

Once the optimal operation and number of KAN heads are determined, we experiment with different combinations of pre-training strategies in our proposed TFKT V2. Table 8 compares the performances among different variants of pre-training EfficientNet and Swin Transformer in settings A and B performances are compared in Table 9. Models 1 to 4, 5 to 8, and 9 to 12 utilized Swin Transformer, Swin-KAN, and Swin-KAT, respectively. In both A and B settings, TFKT V2 with KADID pre-trained EfficientNet and ImageNet pre-trained Swin-KAT achieves the best scores in all four metrics. This demonstrates the effectiveness of natural image pre-training of EfficientNet in combination with the ImageNet pre-training of Swin-KAT. Also, we performed Wilcoxon signed-rank tests across two models in settings A and B (Tables 8 and 9). Between models 4 and 12, the p-value<0.001 confirms that our pre-training with CNN and fine-tuning with Swin-KAT are significantly better than model 4. Also, between models 8 and 12, the p-value is 0.06 (p-value>0.05); however, these achieve an overall score of 2.6890 versus 2.6975 and 2.8841 versus 2.8890, indicating performance improvement.

**Table 8:** Ablation study based on quantitative evaluation of model performance with various combinations of EfficientNet [with or without natural image (KAD) pre-training] and modified Swin Transformer [with or without ImageNet (IM) pre-training] to highlight the importance of natural image pre-training for EfficientNet and ImageNet pre-training for the modified Swin Transformer. These experiments are done using the setting A. The best results are bolded.

No.	EfficientNet		Swin Transformer		r	ρ	τ	s
	Without KAD	With KAD	Without IM	With IM				
1	✓	—	✓	—	0.8830	0.8304	0.6321	2.3456
2	✓	—	—	✓	0.9809	0.9840	0.9097	2.8745
3	—	✓	✓	—	0.9794	0.9834	0.9084	2.8712
4	—	✓	—	✓	0.9842	0.9846	0.9126	2.8814
5	✓	—	✓	—	0.9335	0.9563	0.8362	2.7260
6	✓	—	—	✓	0.9847	0.9843	0.9089	2.8778
7	—	✓	✓	—	0.9679	0.9728	0.8808	2.8215
8	—	✓	—	✓	0.9857	0.9850	0.9134	2.8841
9	✓	—	✓	—	0.9202	0.9405	0.7982	2.6589
10	✓	—	—	✓	0.9820	0.9826	0.9093	2.8739
11	—	✓	✓	—	0.9852	0.9843	0.9097	2.8792
12	—	✓	—	✓	**0.9858**	**0.9861**	**0.9171**	**2.8890**

**Table 9:** Ablation study based on quantitative evaluation of model performance with various combinations of EfficientNet [with or without natural image (KAD) pre-training] and modified Swin Transformer [with or without ImageNet (IM) pre-training] to highlight the importance of natural image pre-training for EfficientNet and ImageNet pre-training for the modified Swin Transformer. These experiments were performed using the testing set environment (setting B). The best results are bolded.

No.	EfficientNet		Swin Transformer		r	ρ	τ	s
	Without KAD	With KAD	Without IM	With IM				
1	✓	—	✓	—	0.8555	0.7676	0.5726	2.1958
2	✓	—	—	✓	0.9221	0.9294	0.7746	2.6261
3	—	✓	✓	—	0.9389	0.9416	0.8007	2.6811
4	—	✓	—	✓	0.9434	0.9425	0.8017	2.6876
5	✓	—	✓	—	0.9119	0.9208	0.7626	2.5953
6	✓	—	—	✓	0.9421	0.9386	0.7961	2.6768
7	—	✓	✓	—	0.9421	0.9386	0.7961	2.6768
8	—	✓	—	✓	0.9434	0.9423	0.8032	2.6890
9	✓	—	✓	—	0.9196	0.9199	0.7548	2.5943
10	✓	—	—	✓	0.9373	0.9382	0.7956	2.6712
11	—	✓	✓	—	0.9427	0.9388	0.7946	2.6761
12	—	✓	—	✓	**0.9462**	**0.9440**	**0.8073**	**2.6975**

#### 4.4.2 Range-wise IQA evaluation

**Fig. 9:**
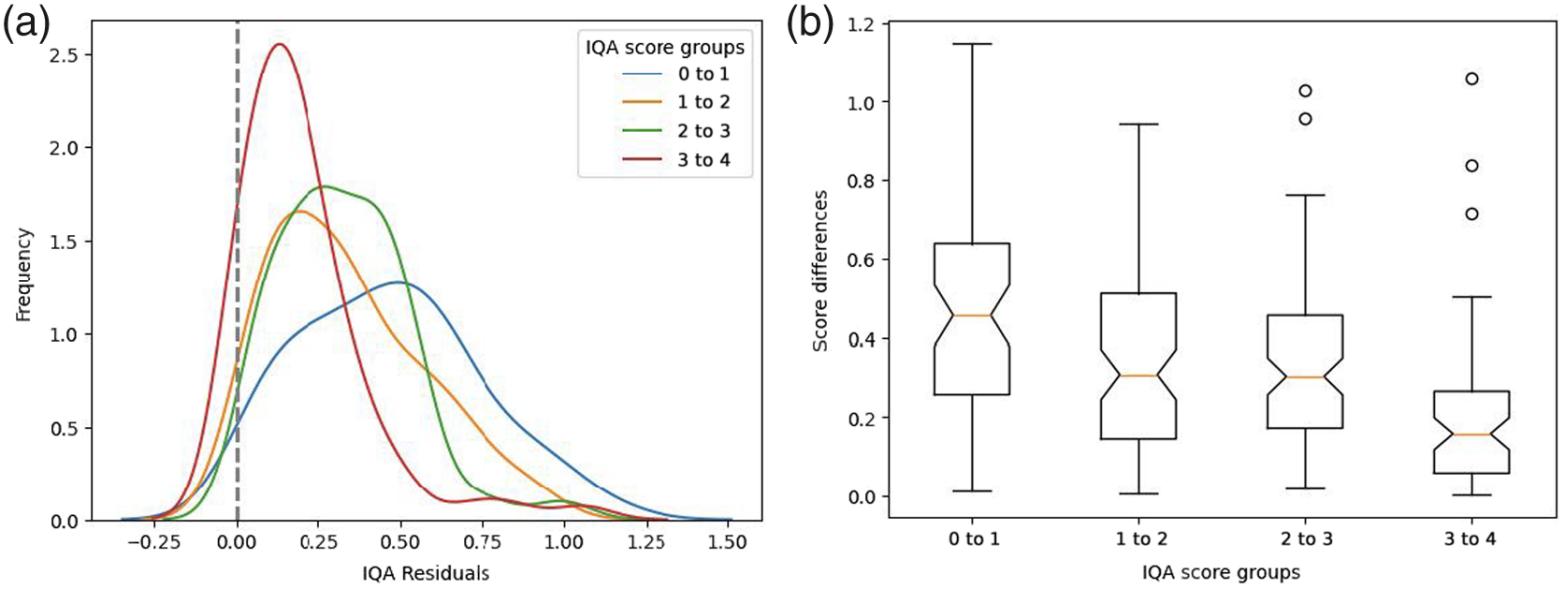
Range-wise evaluation of the proposed TFKT V2 in assessing CT image quality from the LDCTIQA test set. Kernel density plot and box plot illustrate the performances across various IQA groups.

To further analyze the model performance, we grouped the test samples into four different categories based on their reference IQA scores: 0 to 1, 1 to 2, 2 to 3, and 3 to 4. On average, TFKT is ∼86% accurate in predicting the correct IQA group. The 3 to 4 range group is the most accurate (95%), whereas the 0 to 1 group is the least accurate (47%). Figure 9 illustrates the error distributions across the four IQA subsets. As can be seen, the model is more effective for the higher IQA scores. For the 3 to 4 range group, the error distribution is close to normal, indicating a good fit. On the other hand, the distribution deviates from normal for lower IQA score ranges. Similarly, the boxplot suggests better agreement and a more compact error spread for the 3 to 4 IQA range group over the other three. Consistent with this interpretation, the Kruskal–Wallis H-test^41^ shows significant differences in performance for low and high IQA score groups (p-value<0.05). With pairwise testing, we found that there is no significant difference between the 1 to 2 and 2 to 3 IQA score groups (p-value≈0.95). This could primarily be due to the range-wise distribution differences in the two data sources (Mayo Clinic, United States, versus National Cancer Center, South Korea) of the training set.^8^ These insights should guide the development of more sophisticated approaches (e.g., data shift) for future IQA predictions in CT images.

#### 4.4.3 Validation on pediatric CT exam

**Fig. 10:**
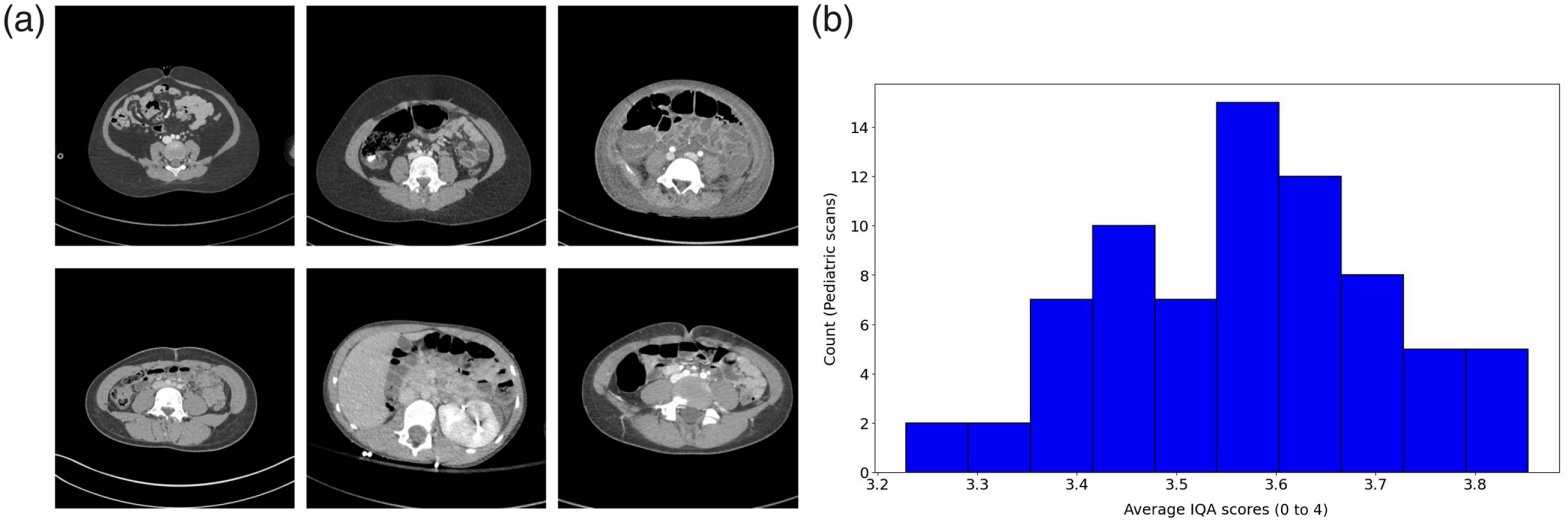
(a) Sample CT images in the pediatric scan dataset (window width: 500; center: 1050). (b) Model predicted IQA scores demonstrate good agreement with the clinical diagnostic quality (IQA score>3) for clinical pediatric abdomen CT images.

We performed an inference of our proposed model on an out-of-domain clinical dataset of 73 abdominal CT scan images acquired from pediatric patients (below 12 years old). Please note that the CT scans were collected with the proper approval of the Institutional Review Board at the University of Kentucky Hospital. As the LDCTIQA dataset only provides slice (2D) image-based scores, we averaged the slice-wise scores to obtain the final IQA score for each of the pediatric scans. Although we do not have reference radiologists-assigned IQA scores available, the predicted average IQA scores as shown in Fig. 10 demonstrate good (diagnostic) quality. This was also validated by two expert abdominal radiologists. We randomly selected 10 scans from the dataset and presented them along with the corresponding predicted IQA scores to the radiologists. Both radiologists confirmed the consistency between the IQA scores and the actual quality of the images presented. The scatter plot presents the model-predicted IQA scores for pediatric CT scans. The average IQA score over 73 patients is 3.572, and the IQA scores range between 3.229 and 3.853. According to the LDCTIQA challenge’s IQA definition, a score of 3 indicates good quality—images are good for diagnostic interpretation. The plot demonstrates a compact distribution of IQA scores with a low variance of 0.021, signifying consistent model performance across patients. The relatively high predicted scores align with expert radiologist validation, indicating that the images possess good diagnostic quality. Pediatric CT scans, by their nature, may yield higher IQA scores. This consistency highlights the robustness of the IQA prediction model and makes the model a reliable tool for diagnostic-quality assessments.

#### 4.4.4 Inference time and memory consumption

We measure the average inference time of 34.35 ms and memory consumption of 1436.09 MB for testing a single slice image using an NVIDIA A4000 GPU. The paper^32^ presents a comparative analysis of various models based on their average inference time and memory consumption for processing a single slice image, and the experiments are conducted using an NVIDIA GeForce RTX 2080 Ti GPU. Although direct comparisons of time and memory consumption can be challenging due to varying experimental setups, our results highlight a significant reduction in inference time. The improved trade-off offered by TFKT V2 makes it more suitable for real clinical practice.

## 5 Conclusions

We have proposed TFKT V2, a no-reference, fully automated, and reliable deep learning–based solution for the CT image quality assessment method. Validating on clinical images from pediatric patients, we have further demonstrated the generalizability of TFKT V2. Our innovative task-specific knowledge transfer can leverage image quality–specific features from natural image pre-training. Exploiting local and global features in a hybrid CNN–transformer model, TFKT V2 is capable of accurately assessing the quality of CT images of arbitrary dose and noise levels. Moreover, the combined MLP and KAN via cross-attention in the Swin Transformer enhances the IQA prediction. An extensive experimentation comparing against a number of baseline and state-of-the-art deep learning-based IQA methods, TFKT V2 is super effective in quantifying CT images of varying noises and artifacts. Finally, TFKT V2 minimizes inference time and enhances feasibility for real-time clinical applications, taking only 34 ms to process a CT image. TFKT V2 performs better compared with the other models because of its task-similar transfer of knowledge. This can help reduce the requirement of a large labeled dataset. Our proposed MK-CA operation within Swin Transformer blocks demonstrates the prediction capability of the model, which enhances feature representation and predictability.

Although the proposed TFKT V2 demonstrates strong performance, some limitations exist. As found in the range-wise evaluation, the model is relatively more accurate for the higher IQA score images than lower score ones. The possible cause is attributed to the difference in IQA scores in data sourced from two distinct racial distributions. Our future work will focus on adopting more informed sampling during training for enhanced generalization. Although our TFKT V2 has a substantial reduction in inference speed compared with the top 5 LDCTIQA challenge algorithms, it has slightly higher memory consumption. This can be overcome by employing some practical parameter-efficient model development techniques.^42^^,^^43^ Our future work will also focus on assessing local image quality with large-scale clinical image datasets from different patient populations across various body regions.

## Data Availability

The 2023 LDCTIQA Challenge dataset can be accessed at https://ldctiqac2023.grand-challenge.org. The pediatric patient data used for additional validation of the proposed model will be available upon request. All our code is publicly available on GitHub which can be accessed at https://github.com/KaziRamisaRifa/TFKT-V2.
